# Supplementary material for: Resilience management during large-scale epidemic outbreaks
Source: Sci Rep. 2018 Jan 30;8:1859. doi: 10.1038/s41598-018-19706-2 (PMC5789872; doi:10.1038/s41598-018-19706-2)
Supplement: Supplementary file 1 — Supplementary Information [file 41598_2018_19706_MOESM1_ESM.pdf]

Resilience management during large-scale epidemic outbreaks

Supporting Information

Emanuele Massaro[a,b,c], **Alexander Ganin**[a,d], **Nicolar Perra**[e,f,g], **Igor Linkov**[a], **and Alessandro Vespignani**[f,g,h]

[a]U.S. Army Corps of Engineers – Engineer Research and Development Center, Environmental Laboratory, Concord, MA, 01742, USA; [b]Senseable City Laboratory, Massachusetts Institute of Technology, 77 Massachusetts Avenue, Cambridge, MA 02139, USA; [c]HERUS Lab, École Polytechinque Fédérale de Lausanne (EPFL), CH-1015 Lausanne, Switzerland; [d]University of Virginia, Department of Systems and Information Engineering, Charlottesville, VA, 22904, USA; [e]Business School of Greenwich University, London, UK; [f]Laboratory for the Modeling of Biological and Socio-Technical Systems, Northeastern University, Boston, MA 02115, USA; [g]Institute for Scientific Interchange, 10126 Torino, Italy; [h]Institute for Quantitative Social Sciences at Harvard University, Cambridge, MA 02138, USA

Reaction process

The SEIR model [1] is customarily used to describe the progression of acute infectious diseases, such as influenza in closed populations, where the total number of individuals in the population is partitioned into the compartments $S(t)$, $E(t)$, $I(t)$ and $R(t)$, denoting the number of susceptible, exposed, infected and recovered individuals at time t, respectively. By definition it follows that $N(t) = S(t) + E(t) + I(t) + R(t)$. In the SEIR model we have three transitions:

$$\begin{aligned} S + I &\xrightarrow{\beta} E + I \\ E &\xrightarrow{\lambda} I \\ I &\xrightarrow{\mu} R \end{aligned} \qquad [1]$$

The first one, denoted by $S \to E$, is when a susceptible individual interacts with an infectious individual and enters in the exposed state with probability β. After a time period (the so-called intrinsic incubation time) $t_i = 1/\lambda$ the exposed individual becomes infected. An infected individual recovers from the disease in the viremic time $t_e = 1/\mu$. The crucial parameter in the analysis of single population epidemic outbreaks is the basic reproductive number R_0, which counts the expected number of secondary infected cases generated by a primary infected individual, given by $R_0 = \beta/\mu$. Here we propose a characterization of a set of prototypical mechanisms for self-initiated social distancing induced by local prevalence-based information available to individuals in the population. We characterize the effects of these mechanisms in the framework of a compartmental scheme that enlarges the basic SEIR model by considering separate behavioral classes within the population (2). In particular the fear of the disease is what induces behavioral changes in the population (3). For this reason we will assume that individuals affected by the fear of the disease will be grouped in a specific compartment SF of susceptible individuals. We consider a mechanism for which people can acquire fear assuming that susceptible individuals will adopt behavioral changes only if they interact with infectious individuals in the same subpopulations. This implies that the larger the number of sick and infectious individuals among one populations, the higher the probability for the individuals that resides in that nodes to adopt behavioral changes induced by awareness/fear of the disease. Moreover we consider the scenario in which we also consider self-reinforcing fear spread which accounts for the possibility that individuals might enter the compartment simply by interacting with people in this compartment: fear generating fear. In this model people could develop fear of the infection both by interacting with infected persons and with people already concerned about the disease. A new parameter $\alpha \geq 0$, is necessary to distinguish between these two interactions. We assume that these processes, different in their nature, have different rates. To differentiate them we consider that people who contact infected people are more likely to be scared of the disease than those who interact with fearful individuals. For this reason we set $0 \leq \alpha \leq 1$. The fear contagion process therefore can be modeled as:

$$S + I \xrightarrow{\beta_F} S^F + I \qquad [2]$$

where in analogy with the disease spread, β_F is the transmission rate of the awareness/fear of the disease. In addition to the local prevalence-based spread of the fear of the disease, in this case we assume that the fear contagion may also occur by contacting individuals who have already acquired fear/awareness of the disease. In other words, the larger the number of individuals who have fear/awareness of the disease among one individual's contacts, the higher the probability of that individual adopting behavioral changes and moving into the class S^F. The fear contagion therefore can also progress according to the following process:

$$S + S_F \xrightarrow{\alpha\beta_F} 2S^F \qquad [3]$$

Then we consider the fact that people with fear have less probability to become infected:

$$S^F + I \xrightarrow{r_b\beta} E + I \qquad [4]$$

with $0 \leq r_b < 1$(i.e. $r_b\beta < \beta$). Moreover we consider the fact that our social behavior is modified by our local interactions with other individuals on a much more rapidly acting time-scale. The fear/awareness contagion process is not only defined by the spreading of fear from individual to individual, but also by the process defining the transition from the state of fear of the disease back to the regular susceptible state in which the individual relaxes the adopted behavioral changes and returns to regular social behavior. We can therefore consider the following processes:

$$S_F + S \xrightarrow{\mu_F} 2S \quad [5]$$

and

$$S_F + R \xrightarrow{\mu_F} S + R \quad [6]$$

Finally the system can be described by the following set of equations:

$$\begin{aligned} d_t S(t) &= -\beta S(t)\frac{I(t)}{N} - \beta_F S(t)\left[\frac{I(t)+\alpha S^F(t)}{N}\right] + \mu_F S(t)\left[\frac{S(t)+R(t)}{N}\right] \\ d_t S^F(t) &= -r_b \beta S^F(t)\frac{I(t)}{N} + \beta_F S(t)\left[\frac{I(t)+\alpha S^F(t)}{N}\right] - \mu_F S(t)\left[\frac{S(t)+R(t)}{N}\right] \\ d_t E(t) &= -\lambda E(t) + \beta S(t)\frac{I(t)}{N} + r_b \beta S^F \frac{I(t)}{N} \\ d_t I(t) &= -\mu I(t) + \lambda E(t) \\ d_t R(t) &= \mu I(t) \end{aligned} \quad [7]$$

The system described by the Equation 7 is reduced to classic SEIR for $\beta_F = 0$.

Definition of the control time T_C

We set the control time T_C as function of the epidemic extinction time T_E for the different model parameters we considered. The control time T_C corresponds to the maximum extinction time T_E for different values of epidemic reproductive number R_0 an be defined.

Fig. S1 shows the epidemic extinction time decreasing the diffusion parameter p for three different values of the epidemic reproduction number R_0. Fig. S1 shows the value of the control time $T_C(R_0)$ used in our experiments in both homogeneous and heterogeneous networks in the diffusion case.

Critical Thresholds

For the SEIR model identify model a critical mobility value p_c, below which the epidemics cannot invade the metapopulation system given by the equation [2]:

$$p_c = \frac{1}{\hat{N}} \frac{\langle k \rangle^2}{\langle k^2 \rangle - \langle k \rangle} \frac{(\mu+\lambda)R_0^2}{2(R_0-1)^2} \quad [8]$$

where $\hat{N}$ represents the average number of individuals in a population. In Fig. S3 we report the minimum valued of the resilience (points) and the theoretical values of the invasion threshold (dotted lines) in both homogenous and heterogeneous networks. The effect of the heterogeneity on the invasion threshold in metapopulation has been previously extensively analysed [3]. In Fig. S3,Fig. S4, Fig. S5 we report the comparison between homogeneous and heterogeneous cases. Here we consider $\lambda = 0.3$ and $\mu = 0.1$.

Self-initiated behavioral changes

Even in this scenario we observed the presence of a critical value of the precaution level β_F after which there is the reduction of the risk in the system (see Figure 3 in the main text). In correspondence of this critical point it is possible to observe non trivial patterns of the system's functionality [4–14]. Indeed the behavioral changes though complicates the dynamics of the model [15]: in particular, within several regions of the parameter space we observe two or more epidemic peaks that produce non-trivial patterns of the system's critical functionality as shown in Fig. S6. This non-trivial behavior can be easily understood. Behavioral change is a self-reinforcing mechanism until it causes a decline in new cases. At this point individuals are lured into a false sense of security and return back to their normal behavior often causing a multiple epidemic peaks as reported in Fig. S7. Some authors believe that a similar process occurred during the 1918 pandemic, resulting in multiple epidemic peaks [16, 17]. In this following example it is possible to observe that before the critical ($\beta_F = 4.3$) point even if all the populations are interested by the disease the extinction time of the disease itself it is lower if compared with the extinction time caused by the multiple peaks caused by the increasing of the precaution level ($\beta_F = 4.3$). However after the transition point the system starts to recover fast also reducing the risk.

The authors contributed to (A) conceive and design the experiments, (B) perform the experiments, (C) write the paper, (D) develop the model, (E) perform the data driven simulations and (F) analyse the data. Emanuele Massaro A, B, C, D, E, F; Alexander Ganin A, B, C, D; Nicola Perra A, B, C, D, E, F; Igor Linkov C, D; Alessandro Vespignani A, C, D.

The authors declare no conflict of interest.

[2]To whom correspondence should be addressed. E-mail: Igor.Linkov@usace.army.mil, a.vespignani@neu.edu

Data-driven simulations: GLEAM

In order to validate the theoretical framework developed, we considered data-driven simulations using the Global Epidemic And Mobility Model (GLEAM) [18]. GLEAM is based on three different data layers (see Ref. [18] for details). In particular,

- The population layer is based on the high-resolution population database of the Gridded Population of the World project by the Socio-Economic Data and Applications Center (SEDAC) that estimates population with a granularity given by a lattice of cells covering the whole planet at a resolution of $15x15$ minutes of arc.
- Mobility Layer integrates short-range and long-range transportation data. Long-range air travel mobility is based on travel flow data obtained from the International Air Transport Association (IATA) and the Official Airline Guide (OAG) databases, which contain the list of worldwide airport pairs connected by direct flights and the number of available seats on any given connection. The combination of the population and mobility layers allows for the subdivision of the world into geo-referenced census areas obtained by a Voronoi tessellation procedure around transportation hubs. These census areas define the subpopulations of the metapopulation modeling structure, identifying 3,362 subpopulations centered on IATA airports in 220 different countries. The model simulates the mobility of individuals between these subpopulations using a stochastic procedure defined by the airline transportation data. Short-range mobility considers commuting patterns between adjacent subpopulations based on data collected and analyzed from more than 30 countries in 5 continents across the world. It is modeled with a time-scale separation approach that defines the effective force of infections in connected subpopulations (see Ref. [18] for details). In other words, short-range mobility is considered at equilibrium in the time scale of long-range patterns. Here, we restricted our analysis to the continental US. To this end, we considered both long and short range mobility patterns limited to the continental US.
- Epidemic Layer defines the disease and population dynamics. The infection dynamics takes place within each subpopulation and assumes a compartmentalization that can be defined according to the infectious disease under study and the intervention measures being considered. As done for the other simulations we considered a SEIR model.

We applied the travel restrictions by multiplying the mobility flows by p. However, considering that by construction short-range mobility is encoded in the effective force of infection (in other words in the simulations individuals do not "move" due to short-mobility) we estimate the value of $A(t)$ as:

$$A(t) = p \sum_i \frac{[N_i(t) - I_i(t)]}{N_i(t)} \qquad [9]$$

1. Anderson RM, May RM, Anderson B (1992) *Infectious diseases of humans: dynamics and control.* (Wiley Online Library) Vol. 28.
2. Colizza V, Vespignani A (2008) Epidemic modeling in metapopulation systems with heterogeneous coupling pattern: Theory and simulations. *Journal of theoretical biology* 251(3):450–467.
3. Colizza V, Pastor-Satorras R, Vespignani A (2007) Reaction–diffusion processes and metapopulation models in heterogeneous networks. *Nature Physics* 3(4):276–282.
4. Linkov I et al. (2013) Measurable resilience for actionable policy. *Environmental science & technology* 47(18):10108–10110.
5. Sheffi Y, et al. (2005) The resilient enterprise: overcoming vulnerability for competitive advantage. *MIT Press Books* 1.
6. Cimellaro GP, Reinhorn AM, Bruneau M (2010) Framework for analytical quantification of disaster resilience. *Engineering Structures* 32(11):3639–3649.
7. Cutter SL et al. (2013) Disaster resilience: A national imperative. *Environment: Science and Policy for Sustainable Development* 55(2):25–29.
8. Linkov I et al. (2014) Changing the resilience paradigm. *Nature Climate Change* 4(6):407–409.
9. Vugrin ED, Warren DE, Ehlen MA, Camphouse RC (2010) A framework for assessing the resilience of infrastructure and economic systems in *Sustainable and resilient critical infrastructure systems*. (Springer), pp. 77–116.
10. Baroud H, Ramirez-Marquez JE, Barker K, Rocco CM (2014) Stochastic measures of network resilience: Applications to waterway commodity flows. *Risk Analysis* 34(7):1317–1335.
11. Adger WN, Hughes TP, Folke C, Carpenter SR, Rockström J (2005) Social-ecological resilience to coastal disasters. *Science* 309(5737):1036–1039.
12. Alderson DL, Brown GG, Carlyle WM (2014) Assessing and improving operational resilience of critical infrastructures and other systems. *Stat* 745:70.
13. Barrett CB, Constas MA (2014) Toward a theory of resilience for international development applications. *Proceedings of the National Academy of Sciences* 111(40):14625–14630.
14. Ganin AA et al. (2016) Operational resilience: concepts, design and analysis. *Scientific reports* 6.
15. Perra N, Balcan D, Gonçalves B, Vespignani A (2011) Towards a characterization of behavior-disease models. *PloS one* 6(8):e23084.
16. Hatchett RJ, Mecher CE, Lipsitch M (2007) Public health interventions and epidemic intensity during the 1918 influenza pandemic. *Proceedings of the National Academy of Sciences* 104(18):7582–7587.
17. Markel H et al. (2007) Nonpharmaceutical interventions implemented by us cities during the 1918-1919 influenza pandemic. *Jama* 298(6):644–654.
18. Balcan D et al. (2009) Seasonal transmission potential and activity peaks of the new influenza a (h1n1): a monte carlo likelihood analysis based on human mobility. *BMC medicine* 7(1):1.

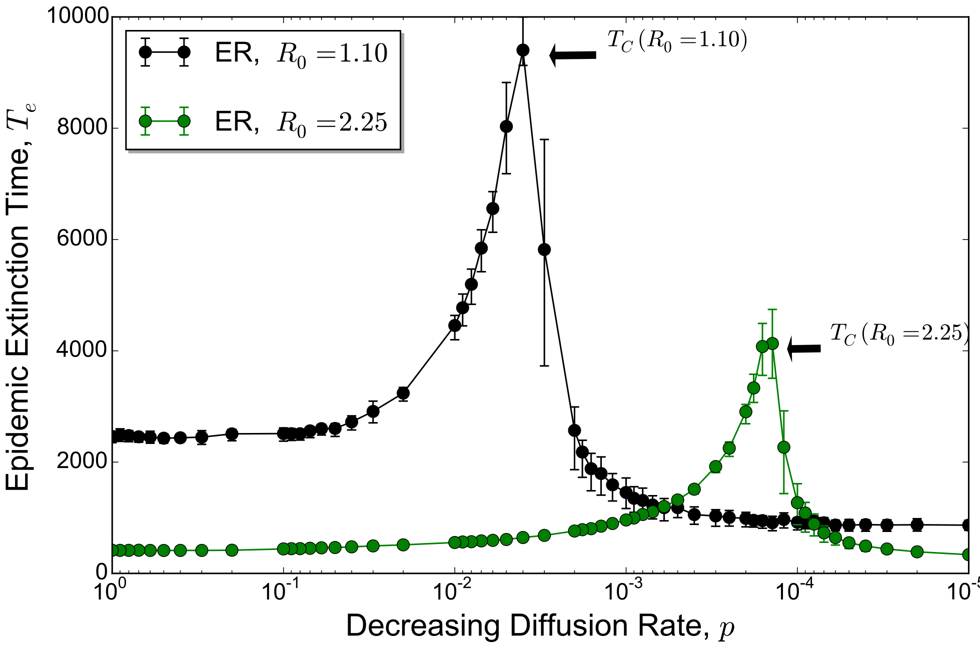

Fig. S1. Control time definition. Median value of the epidemic extinction time T_e as function of the diffusion rate p. The maximum time correspond to the epidemic control time $T_C(R_0)$.

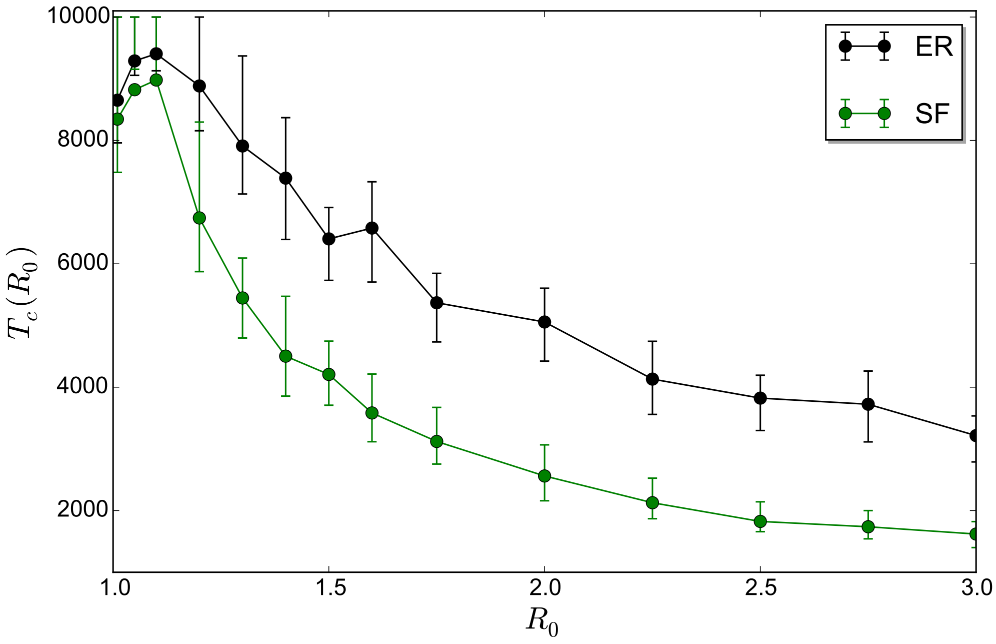

Fig. S2. Different values of the control time in both homogeneous and heterogeneous networks for different values of the epidemic reproduction number R_0.

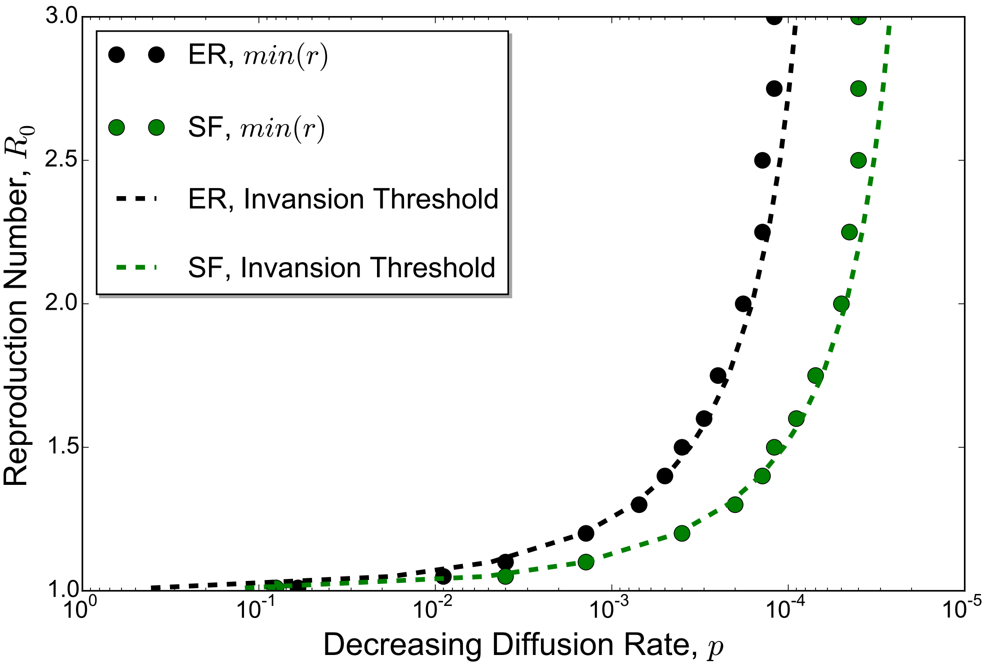

Fig. S3. Effect of the network heterogeneity on the system's risk and resilience. The minimum value of the resilience (dots), which corresponds to the theoretical value of the final fraction of diseased subpopulations D_∞/V at the end of the global epidemic (dotted lines), is shown as a function of the mobility rate p in a homogeneous and heterogeneous networks. The minimum value of the resilience separates the two region of high resilience.

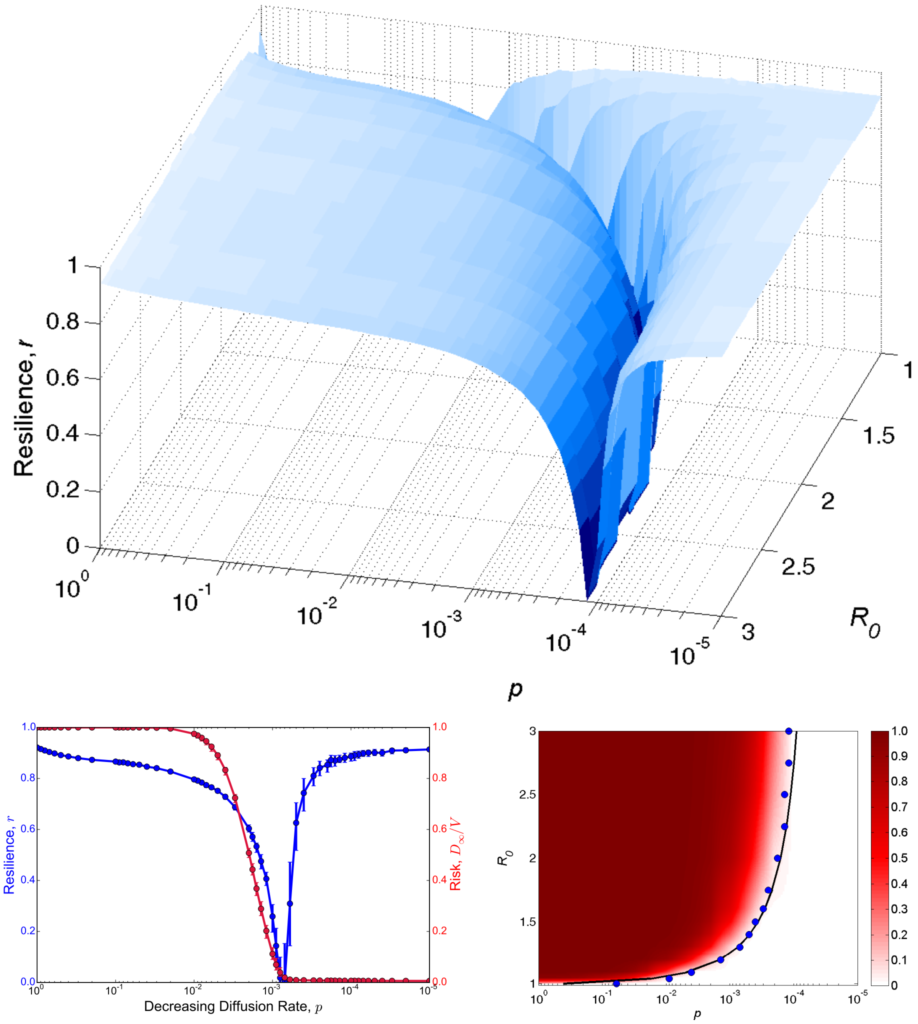

Fig. S4. Resilience surface in homogeneous networks in the plane $(p - R_0)$. Figure B refers to $R_0 = 1.3$.

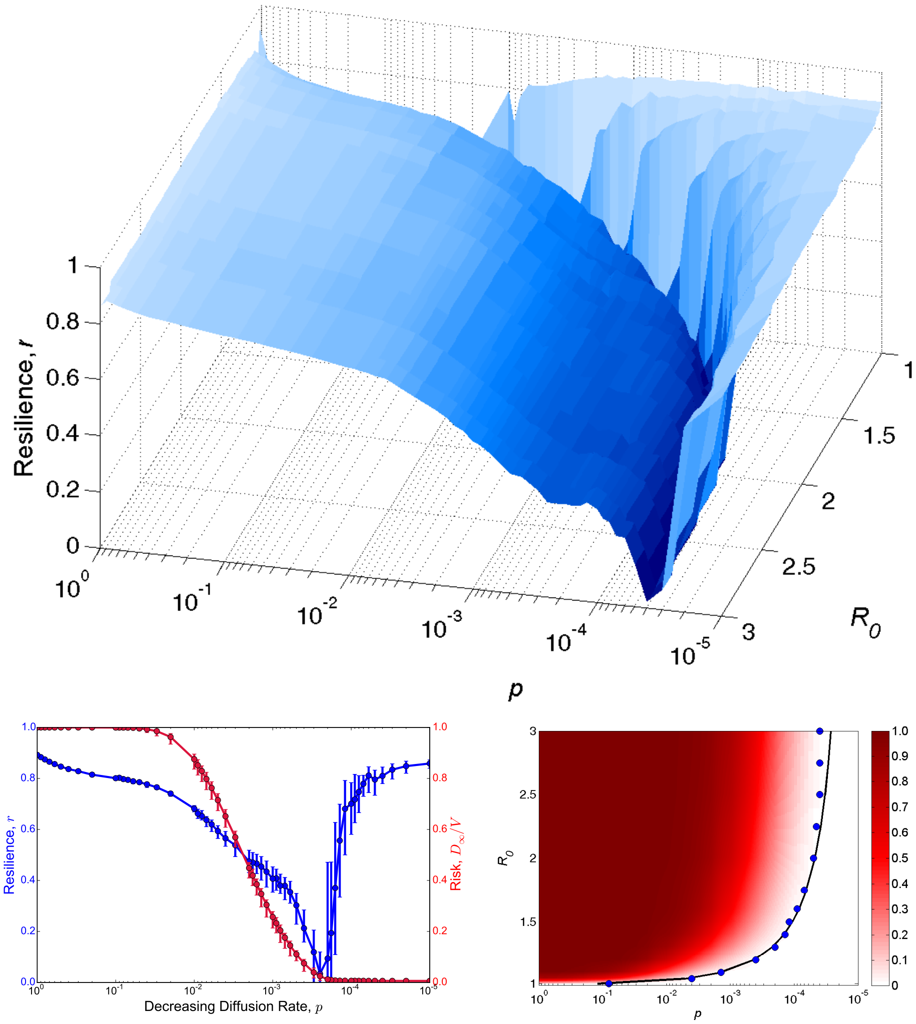

Fig. S5. Resilience surface in heterogeneous networks in the plane $(p - R_0)$. Figure B refers to $R_0 = 1.3$.

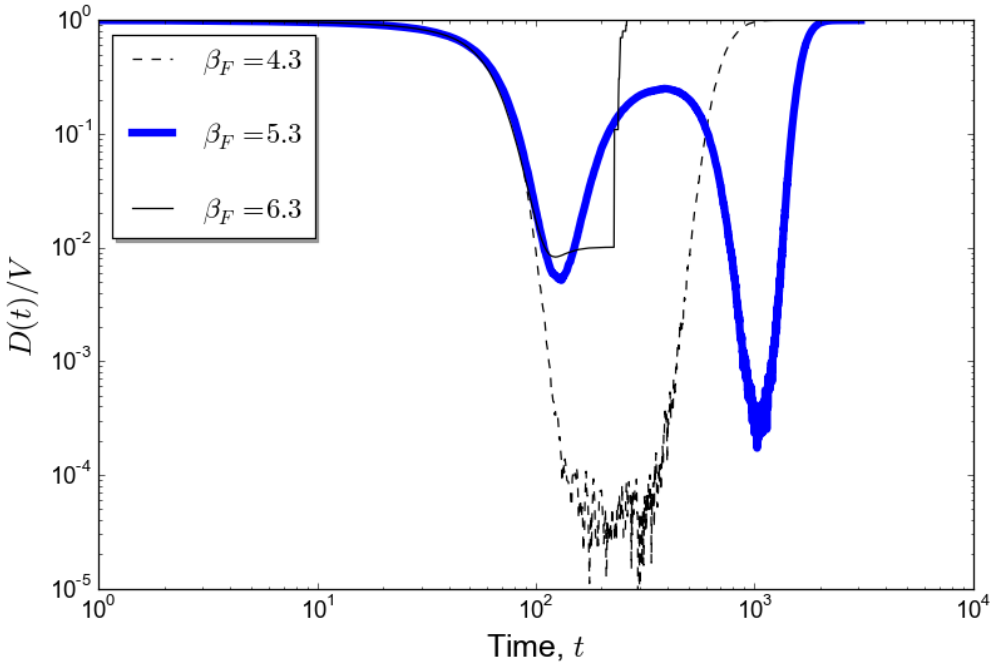

Fig. S6. (log-log) Average values of the system's critical functionality for $R_0 = 2$ before (dotted line), over (red line) and after the transition point.

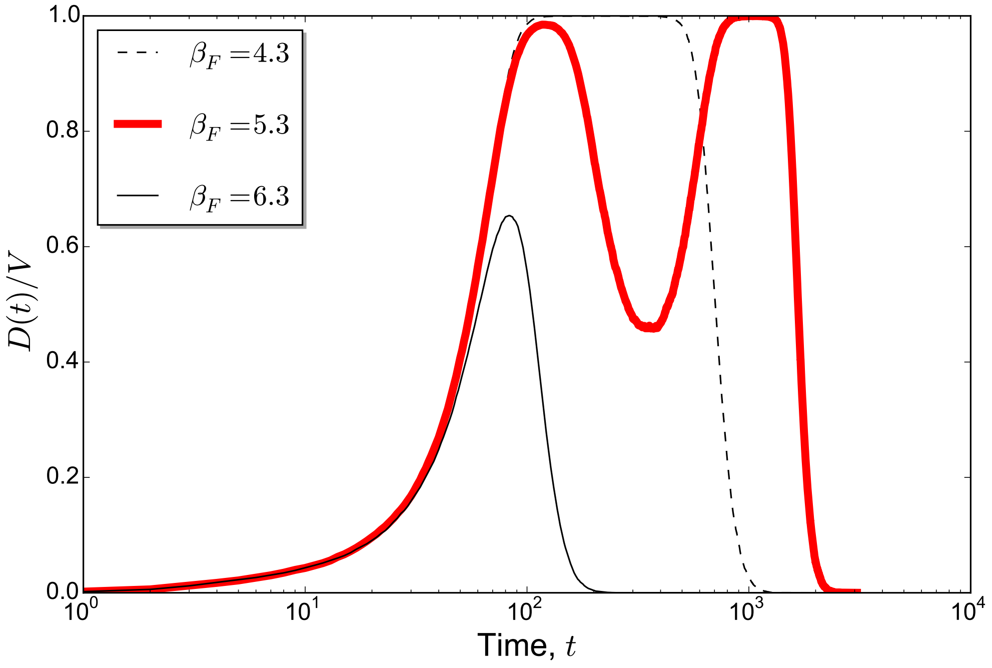

Fig. S7. (log-x) Average values of the diseased populations for $R_0 = 2$ before (dotted line), over (red line) and after the transition point.
